# Meningococcal Sepsis Complicated by Symmetrical Peripheral Gangrene: A Case Report

**DOI:** 10.7759/cureus.9470

**Published:** 2020-07-30

**Authors:** Jessica Ennis, Ola Ahmed, Muhammad Khalid, Patrick A Boland, Michael Allen

**Affiliations:** 1 General Surgery, Connolly Hospital Blanchardstown, Dublin, IRL; 2 Medicine, The Mater Misericordiae University Hospital, Dublin, IRL; 3 Medicine, Connolly Hospital Blanchardstown, Dublin, IRL

**Keywords:** meningococcal sepsis, symmetrical peripheral gangrene, sepsis, dic, amputation

## Abstract

Symmetrical peripheral gangrene (SPG) is a rare complication of septicaemia and disseminated intravascular coagulation (DIC) characterised by distal ischaemia in two or more extremities without large vessel obstruction. SPG has high mortality and morbidity rates, though consensus guidelines for management have yet to be produced.

We herein present the case of a 28-year-old woman with meningococcal septicaemia complicated with extensive bilateral upper and lower limb SPG.

We discuss the various management options of SPG. While reported cases are scarce in the literature, early recognition of sepsis and prompt withdrawal of vasoconstrictors in an intensive care setting, combined with timely heparinisation, appear to be the most common management strategy thus far.

## Introduction

Symmetrical peripheral gangrene (SPG) is a rare complication of septicaemia characterised by distal ischaemia in two or more extremities, without large vessel obstruction [1]. It is an uncommon but severe condition that is usually associated with disseminated intravascular coagulation (DIC), suggesting that DIC may be the final common pathway of its pathogenesis [2]. Peripheries are affected in a symmetrical manner, starting with the lower limbs first and progressing to involve the upper limbs proximally if not reversed [3]. No proper guidelines exist but the current literature suggests that treatment of the underlying aetiology, and discontinuation of vasoconstrictors, if appropriate, should be prioritised [3-4]. Following this, wound care is prioritised by applying supportive measures with optimal dressing application, analgesia, and anti-coagulation in an effort to limit the progression of gangrenous areas. SPG carries a mortality rate as high as 40% in some instances, with most survivors ultimately requiring a life-saving amputation of the affected extremities [3,5]. The aim of this paper is to describe our experience of a unique case of SPG resulting from severe meningococcal septicaemia in a young female patient and expand the knowledge of SPG by collating and reviewing the literature. We also hope to emphasize the diagnostic characteristics and patterns of SPG and discuss future challenges and areas of research. 

## Case presentation

A previously well 28-year-old female patient of Mexican origin presented to the emergency department with a 24-hour history of persistent vomiting, diarrhoea, rigours and epigastric pain. On further questioning, the patient admitted to generalized neck pain in the previous 24 hours but denied photophobia, headaches or rashes. She returned from a trip to Spain five days prior to her presentation and reported no sick contacts before or during her trip.

The patient was lethargic and hemodynamically unstable on arrival - heart rate 138 bpm, blood pressure 64/30 mmHg, respiratory rate 22/min and temperature 37.6 degrees Celsius. Soft epigastric tenderness was demonstrated on physical examination with no guarding or rebound tenderness on deeper palpation. Initial laboratory studies yielded severe metabolic acidosis characterized by an arterial blood gas pH of 7.21 and lactate of 10.5mmol/l. Further laboratory investigations revealed a white blood cell count of 1.7 x109/l, a platelet count of 20 x109/l, C-reactive protein (CRP) level of 374 mg/l, creatinine level of 357 umol/l, urea level of 12.5 mmol/l, international normalized ratio (INR) of 2.6, D dimer of >4400 ng/ml and fibrinogen of 1.27 g/l. The results of an initial portable chest X-ray and dipstick urinalysis were normal. Blood cultures were taken immediately and broad-spectrum antibiotics with fluid resuscitation were promptly commenced. She was urgently transferred to the intensive care unit (ICU) where her condition significantly deteriorated requiring emergency intubation to protect her airway. The patient remained hypotensive, warranting significant vasopressor and inotropic support. She initially required vasopressin 0.04 units/min (2.4 units/hr) that was weaned off over 17 hours, noradrenaline 0.65 mcg/kg/min (40 mcg/min) that was weaned to 0.16 mcg/kg/min by 24 hours, and adrenaline 0.65 mcg/kg/min (40 mcg/min) that was weaned off by 30 hours. The patient was also transfused with four pools of platelets in the first 24 hours.

An initial differential diagnosis of septic shock secondary to meningococcal meningitis was considered based on her recent travel and symptoms, however, a lumbar puncture was contraindicated due to her severe thrombocytopenia.

Peripheral pallor and cyanosis were noted bilaterally in her hands and feet on day one. On examination, her fingertips and feet were cold to touch and her capillary refill time was prolonged at >3-4 seconds. Bilateral radial pulses were present, however, posterior tibial pulses and dorsalis pedis pulses were diminished. At this stage, the suggestion of acute bilateral upper limb and lower limb ischemia secondary to DIC was considered.

At 48 hours, her blood cultures grew meningococcus that was sensitive to ceftriaxone and meropenem, which she was started on, and the public health department was notified as per national guidelines [6]. Prophylaxis was also provided to close contacts and the staff caring for her.

**Figure 1:**
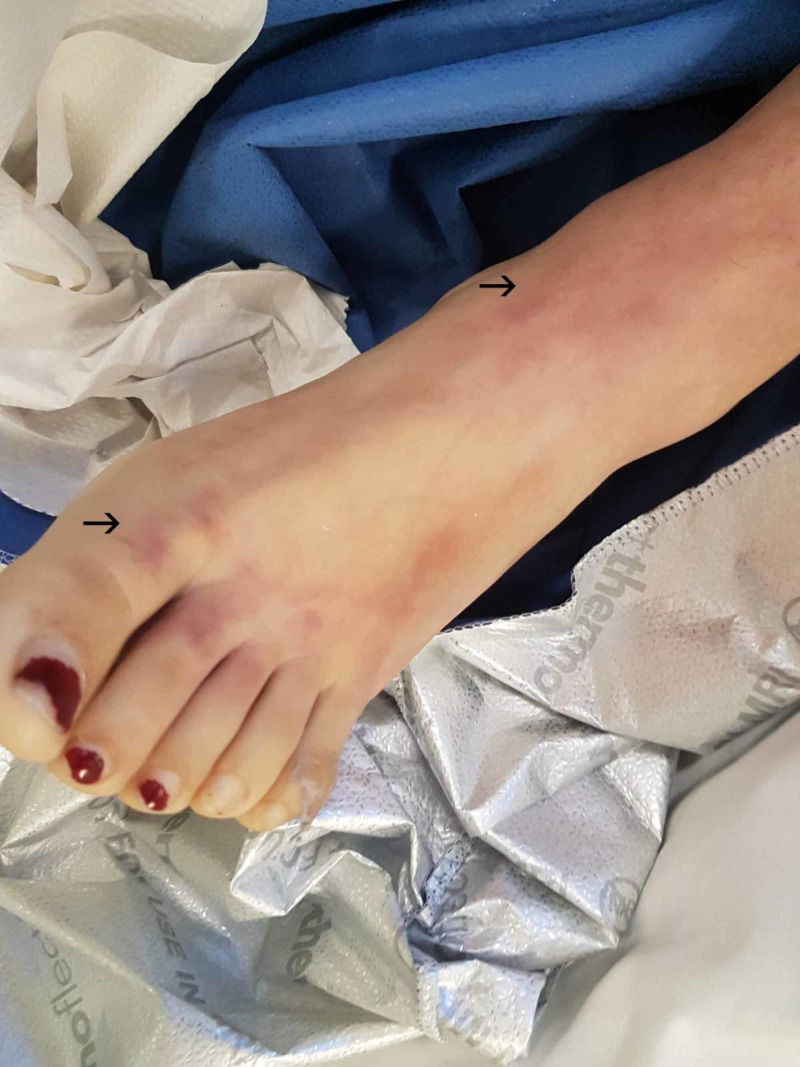
Day 2, dusky violet appearance of the right foot, progressed from pallor and cyanosis the previous day

The bilateral peripheral pallor and cyanosis progressed to a dusky violet colour by day two (Figure 1). Her peripheral pulses were present and confirmed with a handheld bedside Doppler ultrasonography and a diagnosis of impending vascular ischemia was still being considered. The patient’s platelet count remained critical and thus heparinization was still contraindicated at this stage. Vascular surgical consult opted for conservative management and a wait-and-watch policy until the patient’s sepsis had fully resolved.

**Figure 2:**
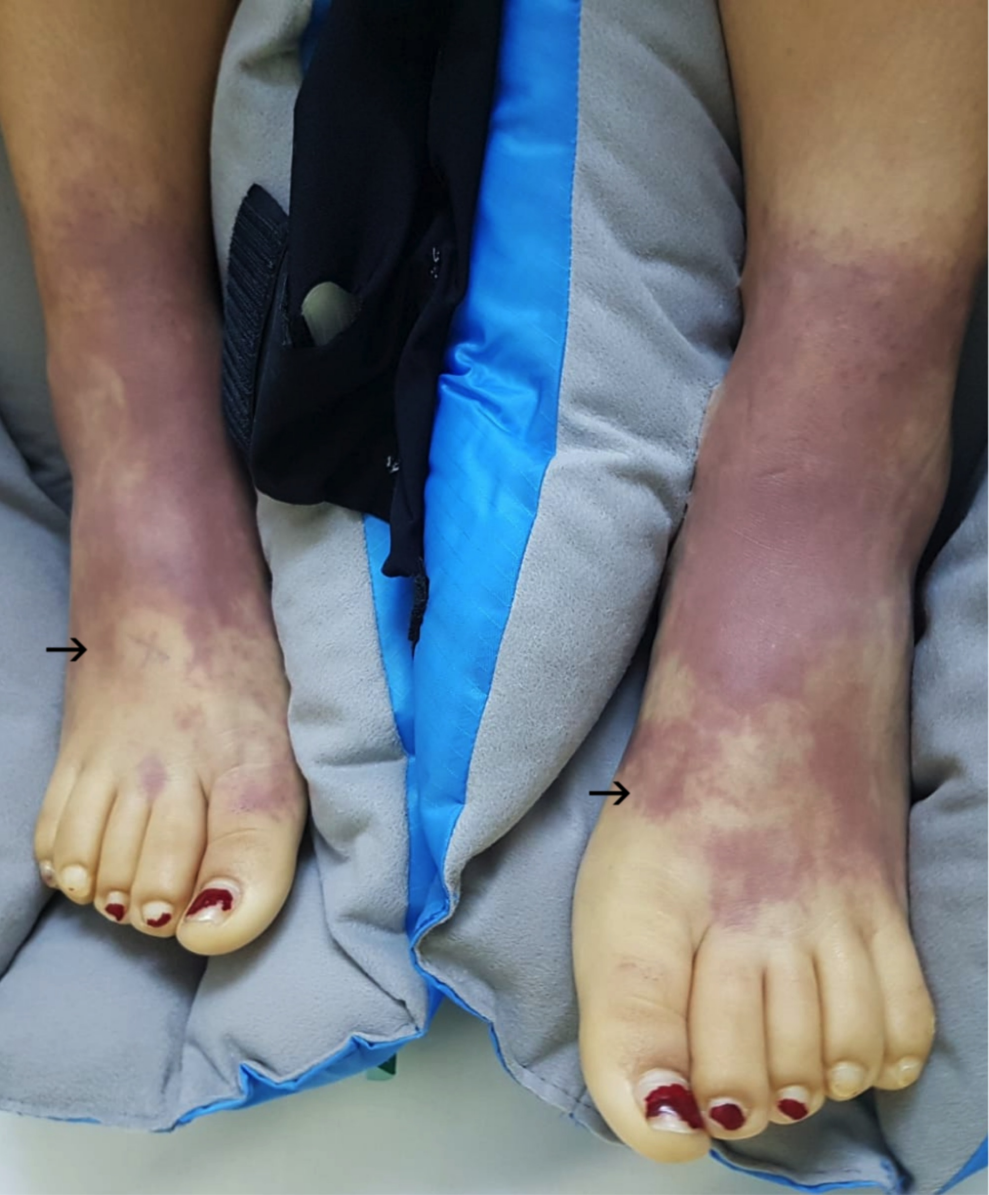
Day 3, bilateral extension of dusky violet colour proximally, with blanching of the digits

**Figure 3:**
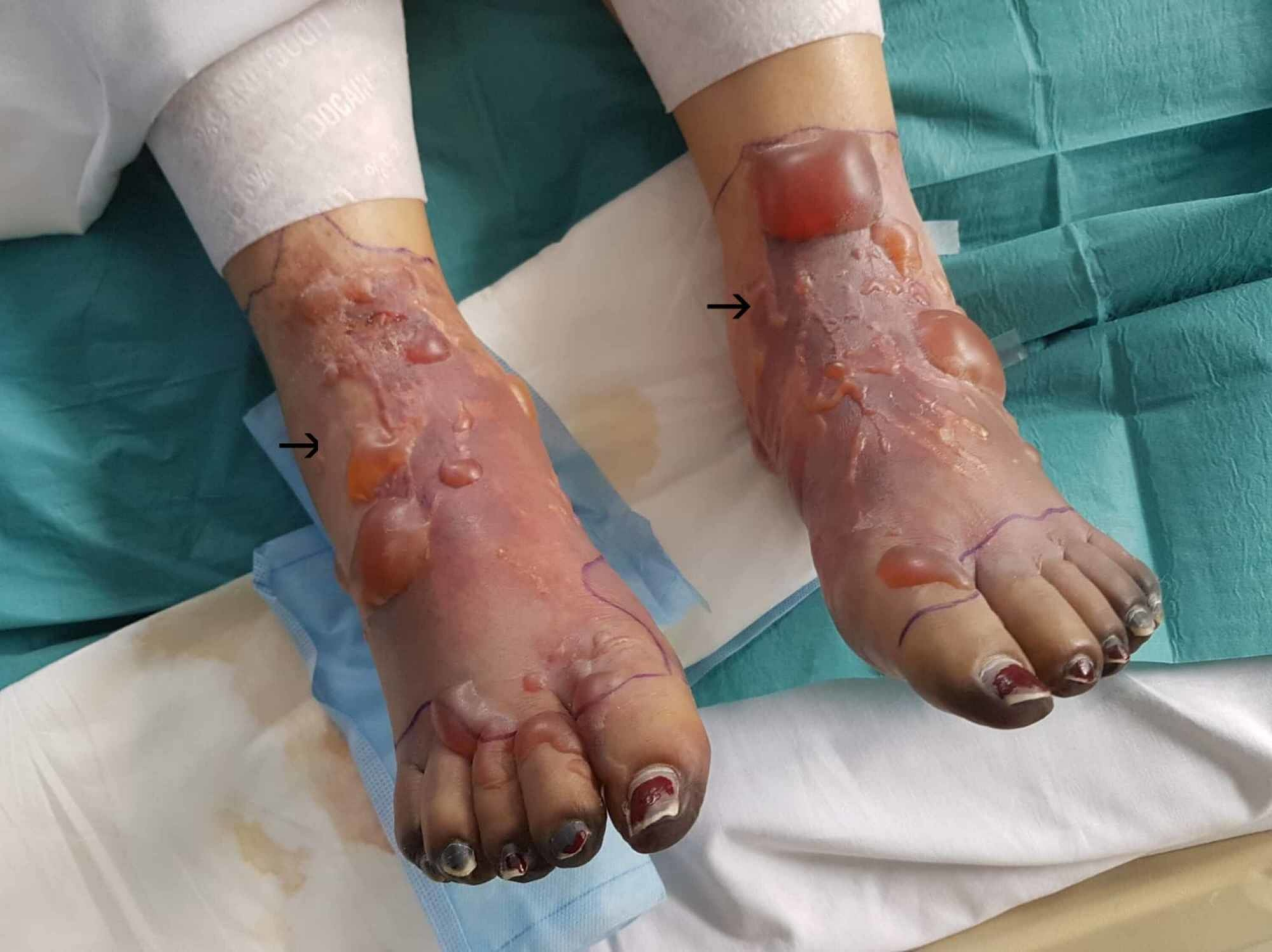
Day 5, vesicles and bullae bilaterally on feet, demarcated areas of dry gangrene at the peripheries of all 10 digits

**Figure 4:**
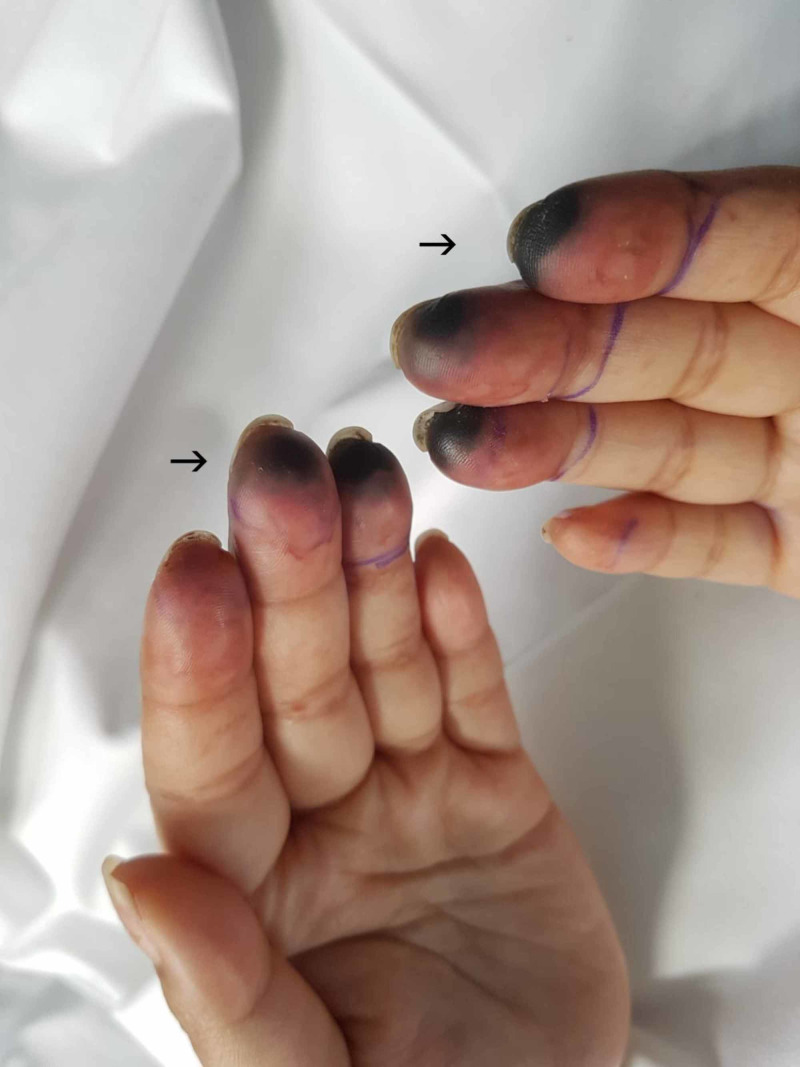
Day 5, demarcated areas of dry gangrene to digits

Her platelet count slowly recovered by day three and continuous venovenous hemofiltration therapy was initiated to manage her persistent acidotic state and unresolving anuria. The patient improved significantly over the course of day three and was weaned from vasopressin. Although still intubated, she became more alert and engaged with staff at times. She began to show signs of severe pain in her upper and lower limbs, with a visible extension of the dusky violet to her limbs (Figure 2). Dialysis was ceased on day four and all inotropic support was weaned by day five. Her peripheries deteriorated further with vesicles and bullae developing on her feet by day five. The tips of all 10 digits of both upper and lower limbs had progressed to demarcated areas of dry gangrene at this stage (Figure 3, Figure 4).

She was extubated on day seven, and by day nine, the patient’s platelets had fully recovered and a conservative treatment plan of neuropathic analgesia, prophylactic low molecular weight heparin and daily dressings was implemented, with optimal pain control by the specialist pain team. 

A spontaneous detachment of her necrotic fingertips occurred with minimal cosmetic and neuropathic injury. Her lower limbs continued to demonstrate significant gangrenous changes with areas of severe skin desquamation and blistering changes that remained warm to touch. The ischemic changes localised to her toes while the rest of her feet improved with healthy pink tissue appearing from the previously blistered areas. The patient remained an inpatient receiving intense physiotherapy, pain management and psychiatric assessment.

**Figure 5:**
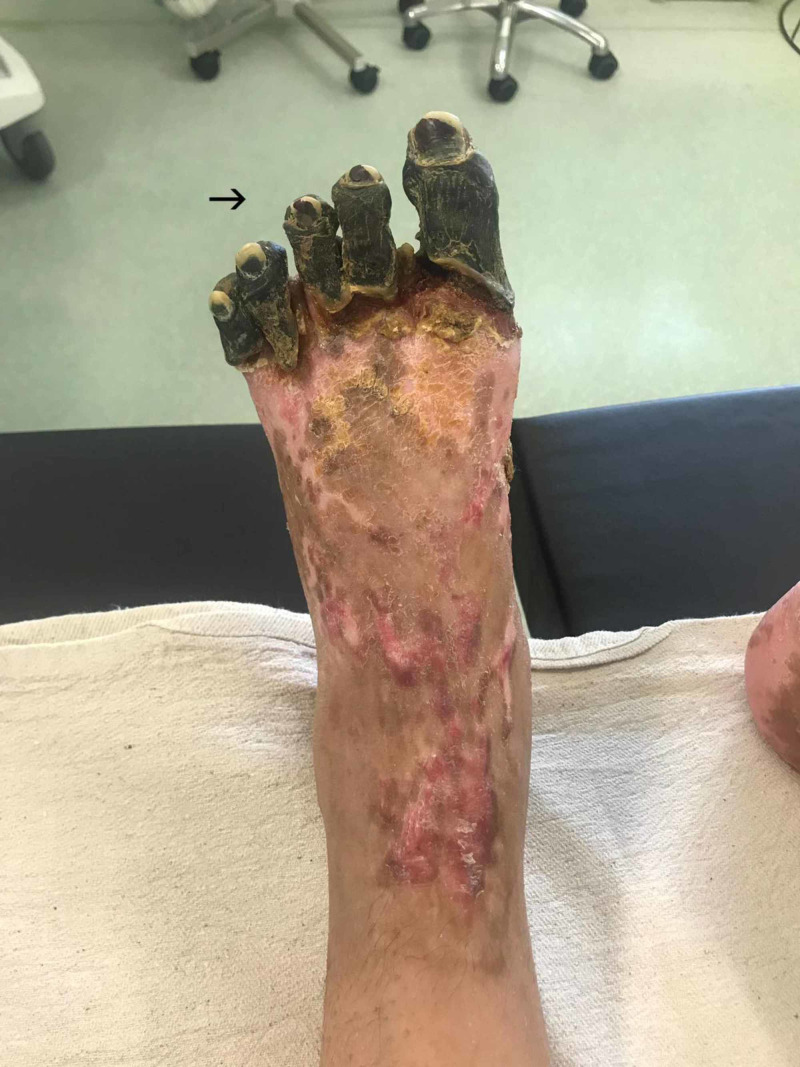
Day 70, well-demarcated dry gangrenous area of all 10 digits on the feet

There was total regression of necrotic tissue on all her fingertips by day 70 and the dry gangrenous area of all 10 digits on the feet had become clearly demarcated and ceased to improve (Figure 5).

**Figure 6:**
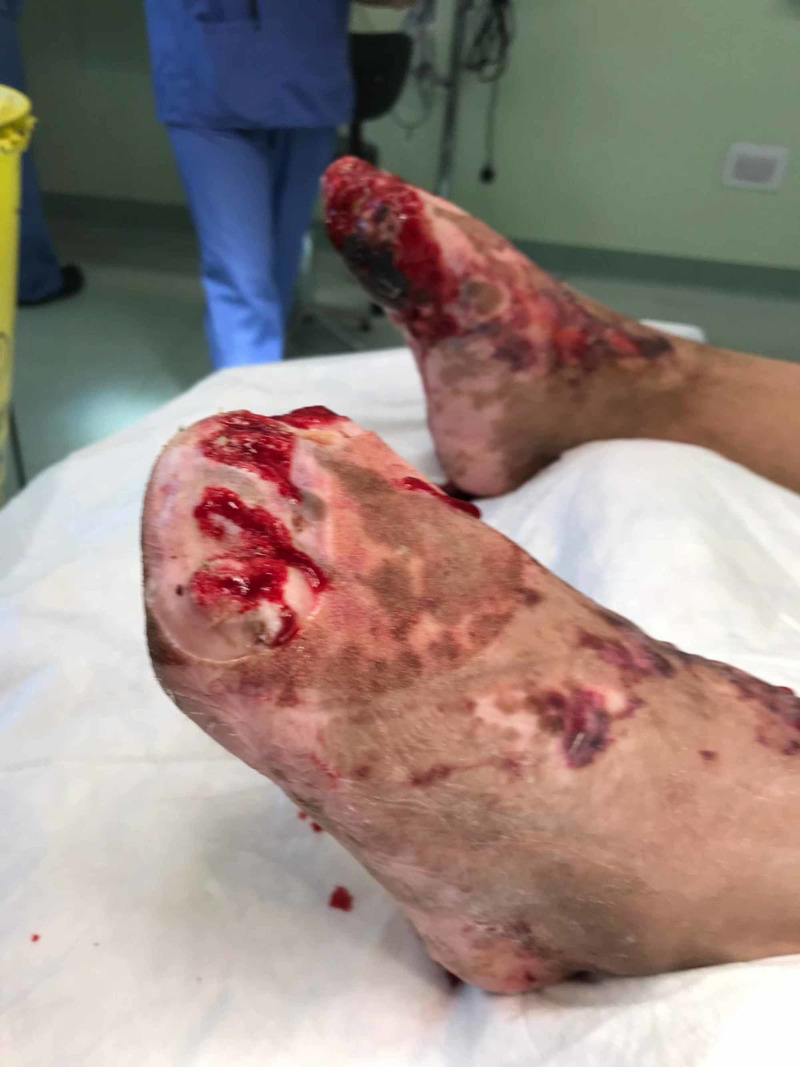
Day 73, immediately post amputation of all toes at the metatarsophalangeal joints

During week 10, the patient underwent an elective bilateral amputation of all the toes at the metatarsophalangeal joints and a vacuum dressing was applied (Figure 6), as her toes were deemed unsalvageable and became malodorous, suggesting a deep soft tissue infection. She required a total of three further wound debridements under general anaesthetic and application of vacuum-assisted closure (VAC) dressing, as this was deemed unsuitable at a ward level at the present time. At seven weeks post-operative, the dressings were changed on a biweekly basis, with weekly input from the plastic surgery team. She continued with extensive physiotherapy and rehabilitation, and following a full resolution of all infection, underwent a reconstructive free flap three months following her initial amputation procedure.

## Discussion

SPG is still, to our knowledge, considered a rare entity, limited to a small collection of case reports and case series in the literature and only a recent review to guide clinicians [3]. SPG has a high reported mortality rate of 40% and an amputation rate of over 70% [3-5,7]. The aetiology of this condition is multifactorial, in our case meningococcal septicaemia was likely the inciting event. Most cases of SPG appear to complicate severe episodes of DIC, likely attributing to an exaggerated inflammatory response, impaired anticoagulant and fibrinolysis causing thrombus formation in small to mid-size vessels [3,8]. Evidence suggests that SPG may occur due to protein C deficiency (seen in severe sepsis, particularly meningococcal septicemia) and reduced procoagulant factor VII (seen in cancer patients, warfarin therapy and heparin-induced thrombocytopenia) [3]. Underlying co-morbidities, low-flow states and vasoconstrictors have also been cited as aggravating factors contributing to disturbed microcirculation [9-11]. The combination of meningococcal septicemia, DIC and use of vasoconstrictors likely paved the way for SPG in this patient.

SPG affects the peripheries symmetrically, classically progressing from the lower limbs proximally to ultimately involve the upper peripheries if not reversed [7,10,12]. Where there is extensive, multicentric, non-acral necrosis present, the term purpura fulminans is used [13]. There is a progressive sequence of pallor, cyanosis, swelling and pain to a dusky violet appearance possibly accompanied by bullae that rapidly leads to dry gangrene as demonstrated in our experience [10,12]. Peripheral pulses remain palpable as the large arteries are not involved. Doppler scans can be helpful in differentiating SPG from other conditions and bone scans can judge if there has been an extension of tissue injury [14].

SPG should be suspected with the first clinical signs, and investigations should be tailored towards the suspected aetiology. This generally includes routine laboratory investigations and imaging, screening for sepsis and DIC. Notably, increased serum lactate levels have been reported to suggest potential SPG [2]. However, no specific biomarkers are pathognomonic and the diagnosis remains largely clinical.

A 2018 review of the treatment of SPG called for a multicenter randomized controlled trial in order to establish detailed guidelines on treatment, as none currently exist [3]. However, it is generally considered that timely discontinuation of vasoconstrictors, prompt management of the underlying aetiology and anticoagulation with low-dose heparin may halt the progression of the disease [2-3,15]. In this case, there was prompt administration of fluids, vasopressin and noradrenaline in order to haemodynamically stabilise the patient at presentation. Empiric antibiotics were also administered early, and she received multiple units of platelets to address her severe thrombocytopenia. The timely tapering of noradrenaline and vasopressin may have contributed to the minimal progression of the gangrene. Prophylactic low molecular weight heparin was also given when she recovered systemically. In this case, there was a total regression of necrotic tissue on her all her fingertips.

There have been case reports of successful treatment with epoprostenol and tissue plasminogen activator infusion, alpha blockade, trimethaphan, sodium nitroprusside and nitroglyceride ointment, however, there have been no trials carried out to support these options [2-3,15].

Early amputation should be avoided to reduce tissue loss [13]. Wounds should be monitored regularly with expert input from tissue viability specialists and both plastic and vascular surgery warranting a multidisciplinary discussion to decide on surgical debridement or amputation [13]. In this case, the decision to amputate was made based on the gangrenous area becoming clearly demarcated from viable tissue and cessation of improvement in this well patient in order to avoid infection.

## Conclusions

SPG has high reported mortality and amputation rates. Even in patients with a relatively good outcome, such as this case, the patient will have a permanent significant disability. Early recognition of this life-altering condition is key to favourable outcomes, especially in the setting of DIC, and with the use of vasoconstrictors, as the early presentation can be as subtle as pallor of the peripheries. This case serves as a reminder of an uncommon but serious complication of sepsis and DIC.

## References

[1] Hayes MA, Yau EHS, Hinds CJ, Watson JD (1992). Symmetrical peripheral gangrene: association with noradrenaline administration. Intensive Care Med.

[2] Sharma BD, Kabra SR, Gupta B (2004). Symmetrical peripheral gangrene. Trop Doct.

[3] Foead AI, Mathialagan A, Varadarajan R, Larvin M (2018). Management of symmetrical peripheral gangrene. Indian J Crit Care Med.

[4] Cartier RA, Tchanque-Fossuo C, Asuku ME, Price LA, Milner SM (2012). Symmetrical peripheral gangrene. Eplasty.

[5] Shimbo K, Yokota K, Miyamoto J, Okuhara Y, Ochi M (2015). Symmetrical peripheral gangrene caused by septic shock. Case Rep Plast Surg Hand Surg.

[6] Centre Centre, H.P.S H.P.S (2019). HPS Centre. Notifiable diseases and their respective causative pathogens. https://www.hpsc.ie/notifiablediseases/listofnotifiablediseases/.

[7] Ghosh SK, Bandyopadhyay D, Ghosh A (2010). Symmetrical peripheral gangrene: a prospective study of 14 consecutive cases in a tertiary-care hospital in eastern India. J Eur Acad Dermatol Venereol.

[8] Molos MA, Hall JC (1985). Symmetrical peripheral gangrene and disseminated intravascular coagulation. Arch Dermatol.

[9] Maheshwari PN, Okwi N, Pore AP (2019). Symmetrical peripheral gangrene of all four limbs: an unusual complication of ureteroscopy. Indian J Urol.

[10] Jiang JL, Tseng LW, Chang HR (2017). Symmetrical peripheral gangrene in sepsis after treatment with inotropes. Ci Ji Yi Xue Za Zhi.

[11] Joynt G, Doedens L, Lipman J, Bothma P (1996). High-dose adrenaline with low systemic vascular resistance and symmetrical peripheral gangrene. S Afr J Surg.

[12] Parmar MS (2002). Symmetrical peripheral gangrene: a rare but dreadful complication of sepsis. CMAJ.

[13] Warkentin TE (2015). Ischemic limb gangrene with pulses. N Engl J Med.

[14] Hamdy RC, Babyn PS, Krajbich JI (1993). Use of bone scan in management of patients with peripheral gangrene due to fulminant meningococcemia. J Pediatr Orthop.

[15] Tripathy S, Rath B (2010). Symmetric peripheral gangrene: catch it early!. J Emerg Trauma Shock.

